# Cerebral venous sinus thrombosis after adenovirus-vectored COVID-19 vaccination: review of the neurological-neuroradiological procedure

**DOI:** 10.1007/s00234-022-02914-z

**Published:** 2022-02-19

**Authors:** Matthias Wittstock, Uwe Walter, Erik Volmer, Alexander Storch, Marc-André Weber, Annette Großmann

**Affiliations:** 1 Department of Neurology, Rostock University Medical Centre, Gehlsheimer Str. 20 18147 Rostock, Germany; 2 Institute for Diagnostic and Interventional Radiology, Paediatric Radiology and Neuroradiology, Rostock University Medical Centre, Rostock, Germany

**Keywords:** Cerebral venous sinus thrombosis, Adenovirus-vectored COVID-19 vaccination, Vaccine-induced immune thrombotic thrombocytopenia

## Abstract

Cerebral venous and sinus thrombosis (CVST) after adenovirus-vectored COVID-19 ChAdOx1 nCov-19 (Oxford–AstraZeneca) and Ad26.COV2.S (Janssen/Johnson & Johnson) is a rare complication, occurring mainly in individuals under 60 years of age and more frequently in women. It manifests 4–24 days after vaccination. In most cases, antibodies against platelet factor-4/polyanion complexes play a pathogenic role, leading to thrombosis with thrombocytopenia syndrome (TTS) and sometimes a severe clinical or even fatal course. The leading symptom is headache, which usually increases in intensity over a few days. Seizures, visual disturbances, focal neurological symptoms, and signs of increased intracranial pressure are also possible. These symptoms may be combined with clinical signs of disseminated intravascular coagulation such as petechiae or gastrointestinal bleeding. If TTS-CVST is suspected, checking d-dimers, platelet count, and screening for heparin-induced thrombocytopenia (HIT-2) are diagnostically and therapeutically guiding. The imaging method of choice for diagnosis or exclusion of CVST is magnetic resonance imaging (MRI) combined with contrast-enhanced venous MR angiography (MRA). On T2*-weighted or susceptibility weighted MR sequences, the thrombus causes susceptibility artefacts (blooming), that allow for the detection even of isolated cortical vein thromboses. The diagnosis of TTS-CVST can usually be made reliably in synopsis with the clinical and laboratory findings. A close collaboration between neurologists and neuroradiologists is mandatory. TTS-CVST requires specific regimens of anticoagulation and immunomodulation therapy if thrombocytopenia and/or pathogenic antibodies to PF4/polyanion complexes are present. In this review article, the diagnostic and therapeutic steps in cases of suspected TTS associated CSVT are presented.

**Supplementary Information:**

The online version contains supplementary material available at 10.1007/s00234-022-02914-z.

## Introduction

Reporting of several fatal cases with cerebral venous sinus thrombosis (CVST) and other thromboses at various sites in combination with a thrombocytopenia 4 to 28 days after vaccination with the SARS-CoV-2 vaccines ChAdOx1 nCov-19 (Oxford–AstraZeneca) and Ad26.COV2.S (Janssen/Johnson & Johnson) led to restriction of vaccination in several countries. This condition has been introduced as vaccine-induced thrombotic thrombocytopenia (VITT) or vaccine-induced postthrombotic immune thrombocytopenia (VIPIT) [1, 2]. More recently, according to the Brighton Collaboration, a more actual definition of thrombosis with thrombocytopenic syndrome (TTS) was proposed, which relies on evidence of thrombosis and new-onset thrombocytopenia without known exposure to heparin [3]. Clinical characteristics and outcome of patients with CVST with TTS (CVST-TTS) and CVST without TTS after SARS-CoV2-vaccination have been described in a recent cohort study. In patients with CVST-TTS, a high mortality at discharge of 47% has been shown [4], even though the mortality is decreasing provided the earlier recognition and improved treatment [5].

Headache is the leading symptom of CVST as well as of CVST-TTS. Therefore, an increasing requirement of MRI examinations of the cranium (cMRI) has been recognized. For avoidance of under- or overdiagnosis, a coordination of the diagnostic procedure between neurologists and neuroradiologists is required. In this review, we aim to summarize the current available literature on the diagnostic management of CVST-TTS. Furthermore, we propose an interdisciplinary agreed diagnostic and therapeutic approach, if vaccine-induced CVST with or without TTS is suspected with reference to the recommendations of the German Society for Thrombosis and Haemostasis Research (GTH) [2].

## History

Thrombotic thrombocytopenia after vaccination has been described occasionally for vaccination against influenza, rabies, and H1N1 [6]. However, CVST was not reported in these patients. Since the beginning of 2020, the COVID-19 pandemic has been keeping the countries in Europe and worldwide in suspense. In the European Union (EU) so far, the European Medicines Agency (EMA) approved two RNA vaccines BNT162b2 (BioNTech/Pfizer) and mRNA-1273 (Moderna) and the two adenovirus-vectored vaccines ChAdOx1 nCov-19 (Oxford–AstraZeneca) and Ad26.COV2.S (Janssen/Johnson & Johnson). With a good effectiveness and safety profile, serious side effects, such as acute severe allergic reactions, are very rare [7]. However, since March 2021, there has been an increase in individual cases with some reported fatal outcomes of CVST-TTS after vaccination with the AstraZeneca vaccine ChAdOx1 nCov-19 (Vaxzevria®). Until March 16, 2021, in Europe (mainly in Great Britain) more than 20 million people had received the AstraZeneca Vaccine, and during this period the EMA registered only 7 cases of multiple vein thrombosis in association with a disseminated intravascular coagulation (DIC) and 18 cases of CVST-TTS. At the same time period, in Germany more than 4.6 million AstraZeneca vaccine doses had been administered and 45 cases with CVST-TTS, some of them with fatal outcome, were reported [8]. Furthermore, additional cases of TTS have been observed with Ad26.COV 2-S vaccine [4, 6, 9].

## Epidemiology

The number of reported cases in Germany associated with dangerous CVST-TTS after ChAdOx1 nCov-19 vaccination was 45 by May 2021 [8]. Considering the number of approx. 2.1 million persons who received the vaccine between 01/29/2021 and 03/19/2021 in Germany who mostly were under 65 years of age (Robert Koch Institute vaccination quotas), a rate of up to 4.3 cases with CVST-TTS per 100,000 vaccinated persons under 65 years of age is estimated. In comparison with the incidence of spontaneous CVST (about 0.22 to 1.75/100,000 person-years based on data from several European countries [10–12]), this indicates a relatively increased risk of CVST-TTS. So far, most of them were women up to 63 years of age (rarely older) [1, 2, 13–16]. Beside, or in combination with extensive (sometimes fatal) thromboses of the large intracranial sinuses also cortical cerebral vein thrombosis may occur. A recent international multicentre cohort study revealed a total of 116 patients with post vaccination CVST [4].

## Pathophysiology

CVST-TTS occurred usually within 4–24 days after vaccination with ChAdOx1 nCov-19 (Oxford–AstraZeneca) [2]. CVST-TTS clinically resembles heparin-induced, antibody-induced thrombocytopenia (HIT-2) [17]. Recently, the assumed antibody-mediated autoimmunological process was further clarified [1, 14, 15]. Previously, conspicuously high antibody titres against platelet factor 4 (PF4)/Heparin have been found in COVID-19 sufferers, however without platelet-activating effect [18]. It has been shown that by the adenovirus-based vector vaccine—other than through the SARS-CoV-2 virus itself—platelet-activating antibodies against PF4/polyanion complexes were induced [1, 14, 15]. The vaccines ChAdOx1 and Ad26.COV2 contain replication-incompetent adenoviral vectors, such as chimpanzee ChAdOx1 and human Ad26.COV2·S, respectively. These two factors encode the spike glycoprotein on SARS-CoV-2 [19]. Interactions between the vaccine and platelets or PF4 could play a role in the pathogenesis of TTS. The possible explanation for this phenomenon is that the free DNA in the vaccines could bind to PF4 and trigger these PF4-reactive autoantibodies in the VITT setting [1]. An important observation in vaccine-induced TTS is the preponderance of thrombosis in the cerebral venous sinuses. Although HIT is a prothrombotic condition, it has not been reported to preferentially being present in association with CVST. Moreover, brain-imaging studies of the patients with post-COVID-19 vaccination TTS and CVST have detected a high rate of intracranial bleeding [16].

## Symptoms of vaccine-induced CVST

The neuropsychiatric symptoms of CVST-TTS are similar as compared to spontaneous aseptic CVST without TTS:

- Subacute or acute, mostly holocephalic headache
- Epileptic seizures
- Personality changes, delirium
- Visual disturbances
- Central paresis and/or other focal neurological symptoms
- Quantitative and/or qualitative disturbance of consciousness

A recent multicentre cohort study analyzed characteristics of CVST-TTS in comparison with CVST without TTS. Frequency of symptoms seemed to be comparable, the possible impairment of consciousness seemed be more severe in CVST-TTS [4]. Since vaccine-induced CVST may be associated with TTS resulting in DIC, it is mandatory to search for signs of related bleeding:

- Cutaneous hematomas
- Petechiae
- Persistent secondary bleeding at cutaneous puncture sites
- Gastrointestinal bleeding

## Laboratory diagnostics of vaccine-induced CVST

### General considerations with CVST

Usually, an increase in plasma d-dimer levels is detectable in venous thrombosis of any location. A normal d-dimer value, however, does not exclude a CVST, especially in patients with (1) isolated headache or (2) duration of symptoms for longer than 1 week [20]. Generally, in patients with CVST, it has to be clarified whether acquired thrombosis risk factors are present (tumor disease, infection, dehydration, steroid intake, etc.), and for women additionally administration of oral contraceptives or pregnancy. If the relevance of acquired risk factors is unclear or a combined etiology is suspected, the search for hereditary or immunogenic thrombophilia is recommended [20]:

- Heterozygous or homozygous factor V Leiden mutation (10–25% of cases)
- Heterozygous or homozygous prothrombin mutation G20210A
- Congenital antithrombin deficiency
- Congenital protein C or protein S deficiency
- Persistently increased factor VIII
- Antiphospholipid antibodies
- Hyperhomocysteinemia
- Very rarely dysfibrinogenemia

### General considerations with DIC

A severe, rapidly developing DIC is confirmed by evidence of thrombocytopenia, prolonged partial thromboplastin time (PTT) and prolonged prothrombin time (PT), increased levels of plasma d-dimer (or fibrin degradation products), and decreased fibrinogen plasma level.

### Procedure at suspicion of vaccine-induced CVST

**Fig. 1:**
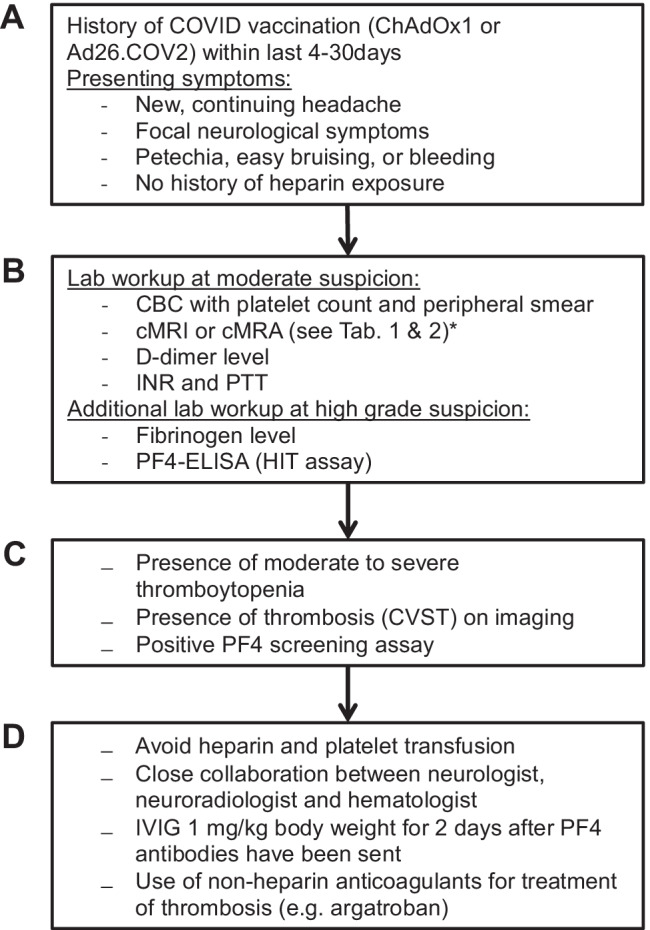
Flowchart if CVST-TTS is suspected. Recommendations according to [31]. **A** Vaccination history and clinical signs of CVST. **B** Graduation of suspicion of CVST-TTS. **C** Diagnostic confirmation. **D** Treatment recommendations in case of CVST-TTS. * hint on MR imaging modalities. *cMRI* cranial magnetic resonance imaging, *cMRA* cranial magnetic resonance angiography, *CBC* complete blood count, INR: international normalized ratio, *PTT* partial thromboplastin time, *PF4* platelet factor 4, *HIT* heparin-induced thrombocytopenia

The laboratory work-up is initially directed by the grading of the clinical suspicion of CVST-TTS: more moderate suspicion or high level of suspicion (Fig. 1). The laboratory work-up at moderate suspicion to be ordered before the indication for cerebral imaging should include the following:

- Blood count (platelet count!)
- Coagulation parameters (d-dimers!)
- Inflammation markers (C-reactive Protein [CRP], leukocytes)

→ At presence of thrombocytopenia: order supplementary test for EDTA-associated pseudothrombopenia and request a manual blood count (with reference to platelet activation). Go on with cranial imaging as specified in the chapters below.

→ At absence of thrombocytopenia: if also plasma d-dimer level is normal, CVST-TTS is unlikely: still, standard MRI with negative MRA or CT with contrast-enhanced venography should be considered. If plasma d-dimer level is increased, MRI with contrast-enhanced venography or CT with contrast-enhanced venography is obligatory.

The laboratory work-up at high suspicion of CVST-TTS or imaging-proven CVST should in addition include the following:

- Screening ELISA for HIT-2 (in many hospitals 24/7)
- Levels of immunoglobulins (exclusion of IgA deficiency prior to potential administration of intravenous immunoglobulins)
- Search for hereditary or immunogenic thrombophilia (see earlier chapter)

→ If HIT-2 screening assay is positive: specialized laboratory workup with heparin-induced platelet aggregation (HIPA) assay and—if HIPA is negative—the modified HIPA assay to detect elevated serum IgG antibodies against PF4-polyanion complexes (“PIPA”—assay, performed in specialized labs, e.g., the Institute of Immunology and Transfusion Medicine Greifswald University Medical Centre); only a positive HIPA or PIPA assay proves vaccine-induced thrombosis [2].

→ If HIT-2 screening assay is positive, CVST is proven on cranial imaging decide urgently on the administration of intravenous Immunoglobulins (Cave: IgA deficiency has to be excluded prior to this therapy! [2].

### Imaging of vaccine-induced CVST

Radiologic imaging in suspected CVST is well established [20]. The procedural imaging approach is not so much different between “regular” CVST and vaccine-induced CVST after vaccination against SARS-CoV-2. Cranial cross-sectional imaging should be performed immediately even in cases of moderate suspicion of CVST on an emergency basis and especially in cases of neurological deficits and conspicuous paraclinical findings (thrombocytopenia, platelet drop, d-dimers—rather unreliable). The choice of method—MRI or CT unenhanced or with a contrast medium—is based on the in-house imaging protocol, local conditions, the patient’s condition, any potential contraindications, and the available neuroradiological expertise. If only unenhanced imaging is performed (in case of moderate suspicion, see above), MRI is the method of choice. An alternative is contrast-enhanced CT. CT and MRI should each be performed with venous angiography (CTA, MRA); native MRA is sufficient in cases of moderate suspicion of CVST (see above) along with normal platelet count and plasma d-dimer level. To avoid radiation exposure, MRI should be preferred in younger patients and during pregnancy. In pregnancy, unenhanced MRA can avoid the administration of contrast medium [21]. In addition, MRI allows clarification of a broader differential diagnostic spectrum and can directly visualize smaller thrombi. We take the knowledge on the typical pathognomonic signs for granted, and provide concise overview in Supplementary Fig. S1. In the diagnosis of cortical venous thrombosis, MRI is superior to CT according to our experience. For this diagnostic question, T2* sequence is more specific in detecting thrombosed veins compared to SWI (susceptibility-weighted imaging) since on SWI both thrombosed and non-thrombosed veins appear with hypointense blooming signal. Here, it is particularly important to also scan the structures close to the vertex. The T2* sequence is highly sensitive as an indirect marker of hemorrhage/blood deposition—and thus also in the context of atypical hemorrhage, possibly in venous outflow obstruction in the context of sinus or venous thrombosis.

The in-house examination protocol on the Siemens Avanto (1.5 Tesla) as well as the Siemens Vida (3.0 T) with corresponding imaging findings may be found in the Supplementary files (see sequences in Supplementary Tables S1, S2, S3).

Until now, at the University Medical Center, we have been able to detect bridging vein thrombosis in 5 patients and atypical bleeding with corresponding thrombi in the sinuses in 2 patients who presented with headache after vaccination with the SARS-CoV-2 vaccines ChAdOx1 nCov-19 (Oxford–AstraZeneca) and Ad26.COV2.S (Janssen/Johnson & Johnson). One course of a patient with CVST-TTS and intracranial bleeding was fatal.

The following three case studies show that after vaccination with ChAdOx1 nCov-19 (Oxford–AstraZeneca) or with Ad26.COV2.S (Janssen/Johnson & Johnson) vaccine with headache, sinus or bridging vein thrombosis should be considered as a possible complication.

#### Case 1

Bridging vein thrombosis after ChAdOx1 nCov-19 (Oxford–AstraZeneca) vaccination 12 days ago.

A 25-year-old female patient was vaccinated with ChAdOx1 nCov-19 (Oxford–AstraZeneca) vaccine. From the 12th day after vaccination, she experienced persistent headache and a feeling of pressure bifrontally—pain scale 6–7 out of 10. This increased especially when turning her gaze. In addition, there was a photo- and phonophobia with staggering dizziness, especially when standing up.

**Fig. 2:**
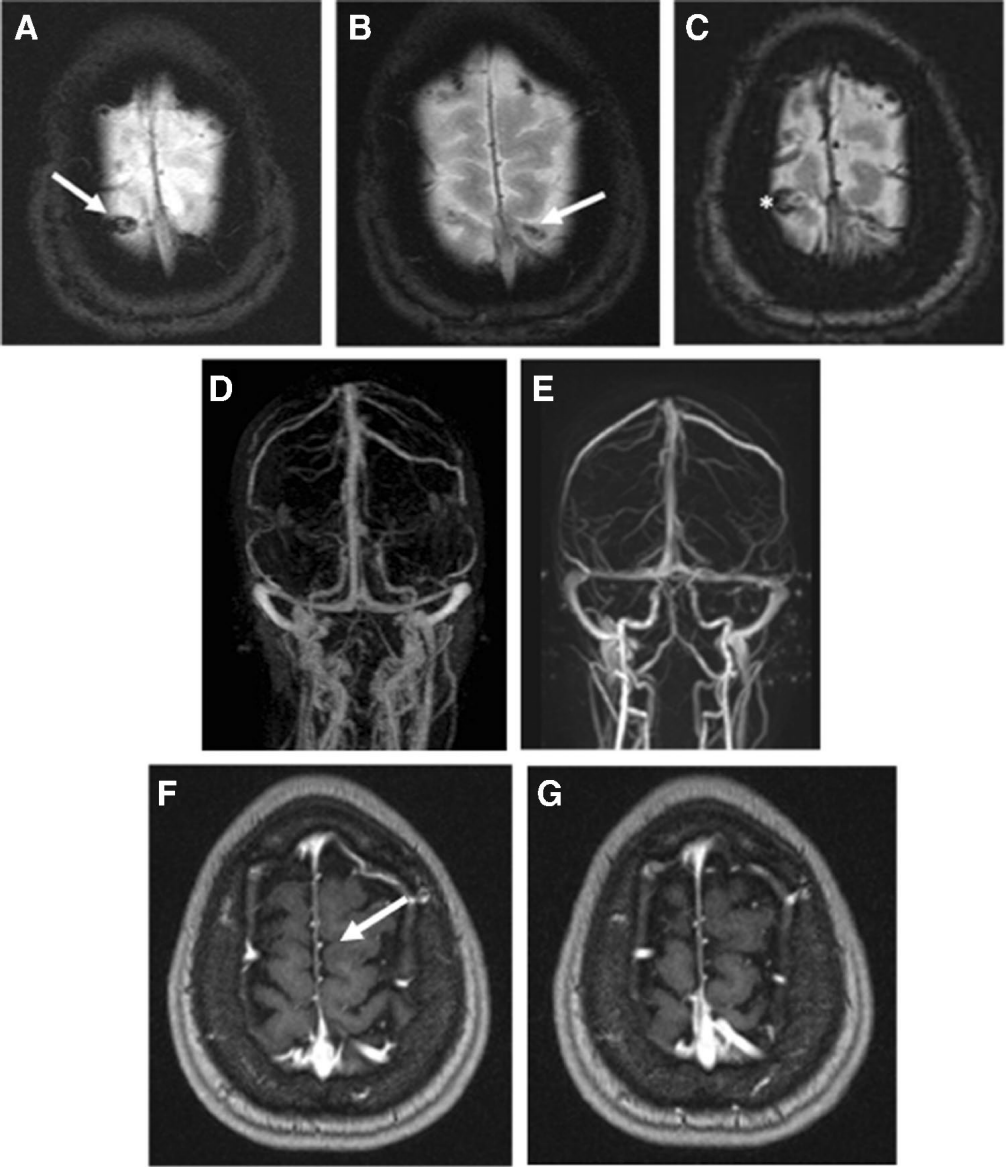
Thrombosis in the area of the cerebral veins bifrontal and bioccipital. **A**, **B** Low signal vascular courses in T2*-weighted imaging (arrows). **C** Blooming sign (asterisk) in susceptibility-weighted imaging (SWI). MRA with absent signaling of the bridging veins on the right (asterisk) in unenhanced PC-MRA (**D**) and CE-MRA (**E**). F, G Multiplanar 3D T1-weighted CE imaging demonstrates lack of contrast of thrombosed bridging veins (arrow)

The typical morphologic correlate of a thrombosis of bridging veins is the “blooming sign” in T2* (see Fig. 2A–C). PC-MRA provided a native method for vascular imaging (see Fig. 2E). Flow artifacts in unenhanced imaging could be verified in CE-MRA (see Fig. 2E) and T1 MPR after contrast agent application (see Fig. 2F, G).

#### Case 2

Bridging vein thrombosis, SAB and thrombosis of the sigmoid sinus after ChAdOx1 nCov-19 (Oxford–AstraZeneca) vaccination 11 days ago.

**Fig. 3:**
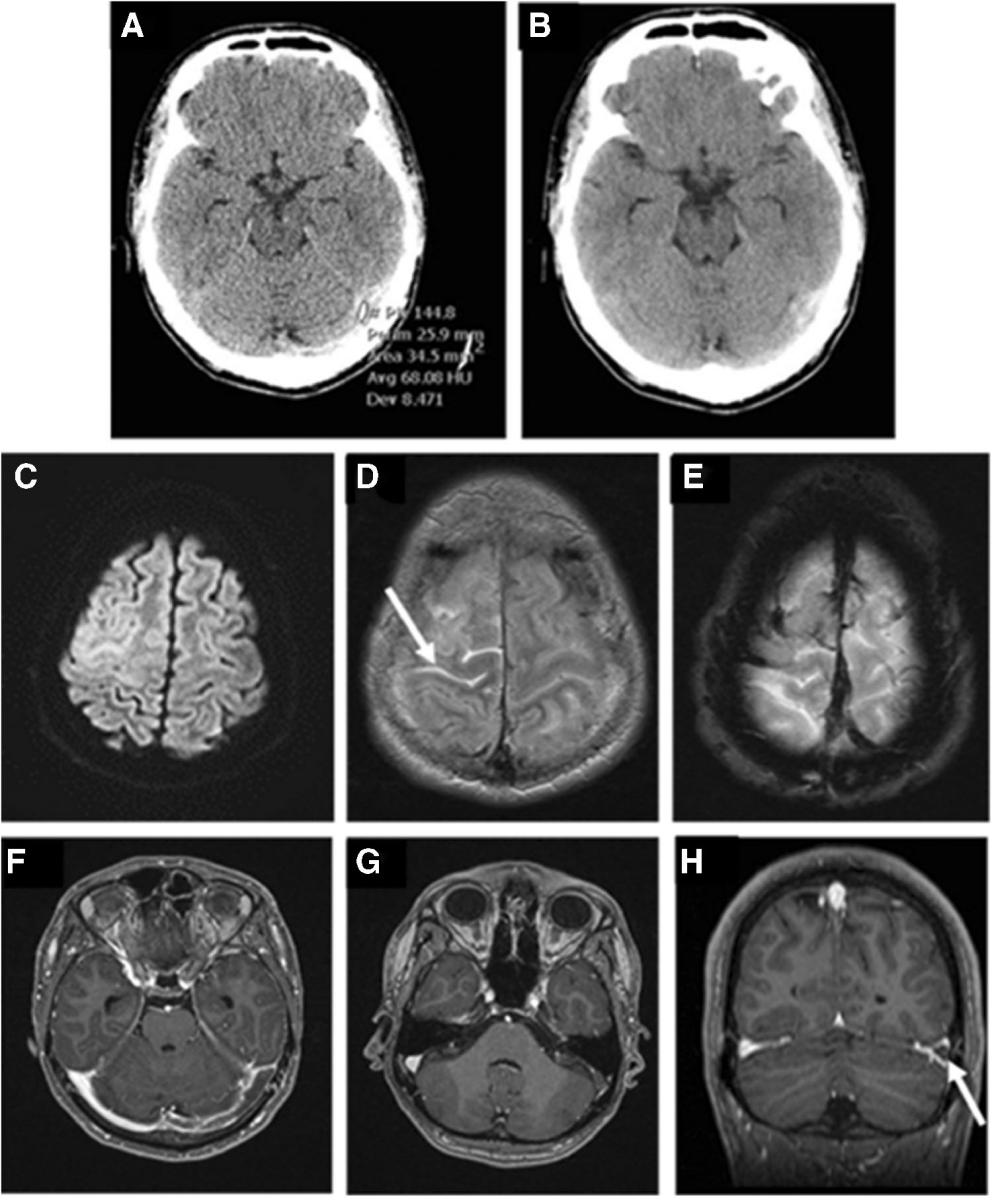
**A**, **B** Unenhanced CT scan. Cord sign of the left sigmoid sinus at an average density of 68 Hounsfield-Units. **C** Hyperintense sulcal diffusion restrictions of the right hemisphere. **D** Corresponding hyperintense SAB (arrow) in FLAIR sequence. **E** Emphasized bridging veins in T2*-weighted imaging. F–H CE T1-weighted images show “triangular sign” of the left sigmoid sinus (arrow)

A 31-year-old man after ChAdOx1 nCov-19-vaccination (11 days ago) showed flu-like symptoms for 1–2 days with improvement. Now cephalgia was present for about 4 days. SARS CoV2-PCR-test was negative. At the time of clinical presentation, he had a holocephalic headache with a range of 8 of 10 in subjective pain scale. Decreasing pain symptoms undergoing intravenous paracetamol application to 6 of 10. The clinical laboratory values show elevated d-dimers and a thrombocytopenia. Figure 3 show left sigmoid sinus thrombosis.

#### Case 3

Atypical bleeding after Ad26.COV2.S (Janssen/Johnson & Johnson) vaccination 12 days ago.

A 29-year-old patient had a Sars-CoV2-vaccination using “one-shot” of Ad26.COV2.S (Janssen/Johnson & Johnson). At the time of his clinical presentation, he was somnolent, disoriented, had headache since 3 o’clock in the night, and recurrent nausea and vomiting. In neurological examination, he had no paresis. The laboratory findings showed evidence of vaccine-induced immune thrombotic thrombocytopenia.

**Fig. 4:**
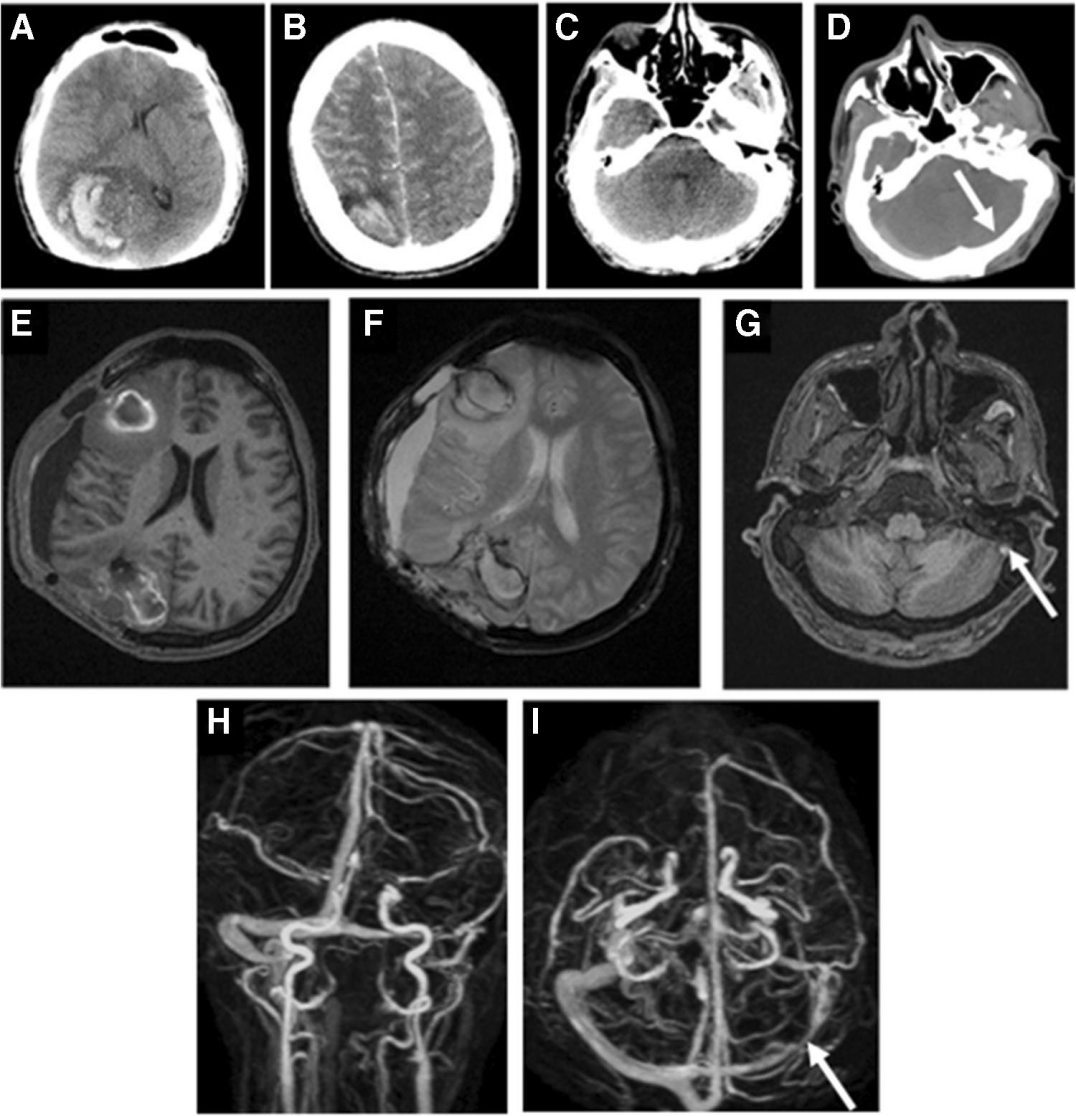
**A**, **B** Unenhanced CT scan with atypical bleeding of the right hemisphere. **C**, **D** Iodine enhanced CT scan shows in side by side comparison thrombosis of the unenhanced left transverse sinus (arrow). Unenhanced MRI after hemicraniectomy and hematoma evacuation. **E** T1-weighted imaging show parenchymal, marginal hyperintense, bleeding spots of the right hemisphere, **F** T2*-weighted images detect the marginal susceptibilities and blood degradation products as well as the perifocal medullary edema. **G** T1-weighted imaging detect the hyperintense thrombotic material within the left sigmoid sinus (arrow). Maximum intensity projections (MIP) of an unenhanced PC-MRI-angiography. **H**, **I** Absent signal in the course of the sinus transversus and sigmoideus on the left side in case of thrombotic occlusion (arrows)

The immediately performed CT and MRI scans revealed atypical hemorrhages of the right hemisphere with thrombosis of the left bridging veins as well as the transverse and sigmoid sinus (Fig. 4).

Hemicraniectomy and hematoma evacuation right hemispheric followed. Indication for anticoagulation with argatroban, a drug used to inhibit blood clotting by direct inhibition of thrombin, was given.

## Therapy of vaccine-induced CVST

### Preclinical considerations

If vaccine-induced CVST with or without TTS is suspected, or if petechial skin hemorrhage is present, there is indication of emergency hospital admission. In the case of a clinically unclear assessment in outpatient setting, the blood count should be checked urgently. In the case of thrombocytopenia, the immediate hospital admission is indicated [22].

### Acute treatment

Because of the potentially foudroyant course, the patient with proven vaccine-induced CVST-TTS should be treated during the first few days on a Stroke unit or, if the course is severe, in the (neuro-) intensive care unit with regular neuromonitoring. The general measures follow the guidelines for therapy for any CVST [20]. The special measures at CVST-TTS are set out below. If CVST-TTS with proven thrombocytopenia is present, the immediate anticoagulation with a HIT2-approved anticoagulant, preferably argatroban or danaparoid, should be started even before receiving the result of the HIT-2 screening assay. If the HIT2 screening assay is negative, a TTS is unlikely. In this case, anticoagulation may be switched to a low molecular weight heparin. In the case of the positive HIT2 screening test, there is a suspicion of a vaccine-induced TTS, and therapeutic anticoagulation has to be continued with argatroban or danaparoid. In addition, the administration of high-dose intravenous immunoglobulins (IVIG) has to be considered [2, 22, 23]. The GTH recommends a IVIG dose of 1 g/kg body weight applied each on day 1 and day 2 (Cave: IgA deficiency has to be ruled out prior to IVIG application; IVIG infusion rate ≤ 10 g/h [2]). The principles of action of IVIG in immunogenic TTS are listed in Table 1 [2, 22, 24]. At lack of response to IVIG plasmapheresis and possibly other immunosuppressive therapies may be considered [25]. In case of a space-consuming brain edema, a decompression craniectomy should be considered [26, 27]. A neuroradiological intervention with slow local fibrinolysis treatment of the intravenous thrombus through arterial side using a microcatheter, optionally in combination with transvenous mechanical recanalization, e.g., by means of a suction catheter, may be considered, however cannot generally be recommended in view to the negative results of the TO-ACT study [26]. On the other hand, we have observed a case of vaccination-induced thrombosis of the internal carotid artery with secondary embolic main stem closure of the middle cerebral artery (without thrombocytopenia) who had a favorable course after systemic thrombolytic therapy [28]. This supports the idea that local fibrinolysis may be a treatment option in desperate cases CVST-TTS. Depending on the course and risk of bleeding (indicated by a normalization of the platelet count), the parenteral anticoagulation may be switched to oral anticoagulation with a vitamin K antagonists (e.g., phenprocuomon) after 1–2 weeks [29]. Alternatively, after the exclusion of antiphospholipid antibody syndrome [30], dabigatran may be considered, especially in patients with mild courses of CVST-TTS [29].

**Table 1:** Effect of intravenous immunoglobulins

CVST-TTS pathomechanism	
Stimulation of the formation of pathogenic antibodies against platelet factor - 4 (PF4)/polyanion complexes (triggered by surface antigen of the viral vector? Triggered by other components of the vaccine?)	
Binding of this anti-PF4 antibody to the FcγIIa receptor on the platelets	
Cross-linking of the Fc receptor by antibody-PF4 polyanion complexes leads to platelet activation	
Thrombocyte activation increases thrombin formation and thus the procoagulatory state	
Effect of IVIG	
Competitive inhibition of the interaction of the anti-PF4 antibodies with the Fc receptor on the platelets	
Thereby reduction of platelet activation and intravascular thrombus formation	
Secondary normalization (re-increase) of the platelet count in the blood	

### Reporting procedures and follow-up care

In Germany, cases of vaccine-induced CVST-TTS have to be reported to the Paul-Ehrlich-Institute (PEI), and as well the pharmaceutical manufacturer should be informed. In addition, there are scientific case collections at national and at European level. For example, all neurological clinics in Germany were asked by the German Society for Neurology (DGN) to include cases of CVST-TTS, intracerebral hemorrhage, or temporal cerebral ischemia related to a COVID-19 vaccination to a national vaccination survey; by mid-April 2021, data of more than 60 patients were evaluated and published promptly [8]. Additionally, the international Cerebral Venous Sinus Thrombosis With Thrombocytopenia Syndrome Study Group reported the clinical characteristics and clinical outcomes of a large cohort of CVST-TTS patients [4]. A second vaccination with an adenovirus-based one vector vaccine after one vaccine-induced CVST-TTS is generally contraindicated. In such a case, a specialist for vaccination should be consulted and a decision on booster vaccination with an mRNA-based vaccine can be made. The duration of oral anticoagulation after vaccine-induced CVST should be set at 4–12 months. Before stopping anticoagulation serum IgG antibodies against PF4/polyanion complexes should be controlled (HIPA assay, PIPA assay, HIT-2 screening ELISA). It can be expected that relevant antibody titres persist over at least about 100 days. Updated recommendations of the medical societies for the acute and long-term management of vaccine-induced CVST should be followed.

## Conclusion

The main symptom of vaccine-induced CVST-TTS is headache beginning 4–24 days after vaccination. First ever seizures, visual disturbances, focal neurological symptoms, and signs of increased intracranial pressure may also occur. If CVST-TTS is suspected, the urgent control of plasma d-dimers level, platelet count, and HIT2 screening assay is mandatory. The imaging method of choice for confirming or excluding CVST is MRI with venous MRA. On T2 *w/SWI sequences, the thrombus causes susceptibility artefacts, in association with pronounced signal cancelation (blooming). MRI/MRA as well as CT/CTA can usually reliably confirm the diagnosis of CVST in synopsis with clinical and laboratory findings. Vaccine-induced CVST-TTS requires specific anticoagulation and immunomodulation therapies, especially in the case of thrombocytopenia and/or the detection of pathogenic antibodies against PF4/polyanion complexes. A close interaction between neurologists, neuroradiologists, and hemostaseology specialists is mandatory.

## Supplementary information

Supplementary Figure S1 Summary of pathognomonic signs of sinus and cerebral venous thrombosis on CT and MRI. A, hyperdense internal cerebral veins on CT scan; B, atypical bleeding on CT scan; C, sulcal SAB, hyperintense in FLAIR, which are hypointense in T2*-weighted images (D); E, blooming of the bridging veins in T2*; “cord sign” in CE-images (F, G); H, missing venous and sinus contrast in CE-MRI. (PNG 837 kb) High resolution image (TIF 260 kb)

Supplementary Table S1 Proposed protocol for MRI in case of suspicion of vaccine-induced CVST (Siemens Avanto; 1.5 T). *DWI* diffusion weighted imaging (diffusions weighted sequence, B1000), *FLAIR* fluid attenuated inversion recovery, *FOV* field of view, *CM* contrast media, *MIP* maximum intensity projection, *MPR* multiplanar reconstruction, *CE*-*MRA* contrast enhanced MR-angiography, *PC* phase contrast (phase-contrast-angiography), *TR* repetition time, *TE* echo time (DOCX 14 kb)

Supplementary Table S2 Proposed protocol for MRI in case of suspicion of vaccine-induced CVST (Siemens Magnetom Vida; 3.0 T). *SWI* susceptibility weighted imaging, *FLAIR* fluid attenuated inversion recovery, *FOV* field of view, *CM* contrast media, *MIP* maximum intensity projection, *MPR* multiplanar reconstruction, *MRA* MR-angiography, *TWIST* time-resolved angiography with interleaved stochastic trajectories, *PC* phase contrast (phase-contrast-angiography), *TR* repetition time, *TE* echo time (DOCX 14 kb)

Supplementary Table S3 Signal of thrombosis in MRI depending on thrombus age (modified [17]). *CM* contrast media, *PCA* phase contrast angiography, *SWI* susceptibility-weighted imaging, *TOF* time-of-flight angiography (DOCX 12 kb)

## Data Availability

Not applicable.

## References

[1] Greinacher A, Thiele T, Warkentin TE, Weisser K, Kyrle PA, Eichinger S (2021). Thrombotic Thrombocytopenia after ChAdOx1 nCov-19 Vaccination. N Engl J Med.

[2] Oldenburg J, Klamroth R, Langer F, Albisetti M, von Auer C, Ay C, Korte W, Scharf RE, Pötzsch B, Greinacher A (2021). Diagnosis and management of vaccine-related thrombosis following AstraZeneca COVID-19 vaccination: guidance statement from the GTH. Hamostaseologie.

[3] Kim D, Robertson JS, Excler JL, Condit RC, Fast PE, Gurwith M, Pavlakis G, Monath TP, Smith J, Wood D, Smith ER, Chen RT, Kochhar S (2020). The Brighton Collaboration standardized template for collection of key information for benefit-risk assessment of nucleic acid (RNA and DNA) vaccines. Vaccine.

[4] Sánchez van Kammen M, Aguiar de Sousa D, Poli S, Cordonnier C, Heldner MR, van de Munckhof A, Krzywicka K, van Haaps T, Ciccone A, Middeldorp S, Levi MM, Kremer Hovinga JA, Silvis S, Hiltunen S, Mansour M, Arauz A, Barboza MA, Field TS, Tsivgoulis G, Nagel S, Lindgren E, Tatlisumak T, Jood K, Putaala J, Ferro JM, Arnold M, Coutinho JM, Sharma AR, Elkady A, Negro A, Günther A, Gutschalk A, Schönenberger S, Buture A, Murphy S, Paiva Nunes A, Tiede A, Puthuppallil Philip A, Mengel A, Medina A, Hellström Vogel Å, Tawa A, Aujayeb A, Casolla B, Buck B, Zanferrari C, Garcia-Esperon C, Vayne C, Legault C, Pfrepper C, Tracol C, Soriano C, Guisado-Alonso D, Bougon D, Zimatore DS, Michalski D, Blacquiere D, Johansson E, Cuadrado-Godia E, De Maistre E, Carrera E, Vuillier F, Bonneville F, Giammello F, Bode FJ, Zimmerman J, d’Onofrio F, Grillo F, Cotton F, Caparros F, Puy L, Maier F, Gulli G, Frisullo G, Polkinghorne G, Franchineau G, Cangür H, Katzberg H, Sibon I, Baharoglu I, Brar J, Payen JF, Burrow J, Fernandes J, Schouten J, Althaus K, Garambois K, Derex L, Humbertjean L, Lebrato Hernandez L, Kellermair L, Morin Martin M, Petruzzellis M, Cotelli M, Dubois MC, Carvalho M, Wittstock M, Miranda M, Skjelland M, Bandettini di Poggio M, Scholz MJ, Raposo N, Kahnis R, Kruyt N, Huet O, Sharma P, Candelaresi P, Reiner P, Vieira R, Acampora R, Kern R, Leker R, Coutts S, Bal S, Sharma SS, Susen S, Cox T, Geeraerts T, Gattringer T, Bartsch T, Kleinig TJ, Dizonno V, Arslan Y (2021) Characteristics and outcomes of patients with cerebral venous sinus thrombosis in SARS-CoV-2 vaccine-induced immune thrombotic thrombocytopenia. JAMA Neurol 78(11):1314–1323. 10.1001/jamaneurol.2021.3619 10.1001/jamaneurol.2021.3619 PMC8479648 34581763

[5] van de Munckhof A, Krzywicka K, Aguiar de Sousa D, Sánchez van Kammen M, Heldner MR, Jood K, Lindgren E, Tatlisumak T, Putaala J, Kremer Hovinga JA, Middeldorp S, Levi M, Arnold M, Ferro JM, Coutinho JM (2022) Declining mortality of cerebral venous sinus thrombosis with thrombocytopenia after SARS-CoV-2 vaccination. Eur J Neurol 29(1):339–344 10.1111/ene.15113 PMC8652752 34536256

[6] Yahyavi-Firouz-Abadi N, Naik RP (2021) Cerebral venous sinus thrombosis associated with vaccine-induced thrombotic thrombocytopenia. Neuroradiol J 19714009211036687 10.1177/19714009211036687 PMC9130610 34333995

[7] Hernández AF, Calina D, Poulas K, Docea AO, Tsatsakis AM (2021). Safety of COVID-19 vaccines administered in the EU: should we be concerned?. Toxicol Rep.

[8] Schulz JB, Berlit P, Diener HC, Gerloff C, Greinacher A, Klein C, Petzold GC, Piccininni M, Poli S, Röhrig R, Steinmetz H, Thiele T, Kurth T (2021). COVID-19 Vaccine-associated cerebral venous thrombosis in Germany. Ann Neurol.

[9] Yocum A, Simon EL (2021). Thrombotic thrombocytopenic purpura after Ad26.COV2-S Vaccination. Am J Emerg Med.

[10] Ferro JM, Correia M, Pontes C, Baptista MV, Pita F (2001). Cerebral vein and dural sinus thrombosis in Portugal: 1980-1998. Cerebrovasc Dis (Basel, Switzerland).

[11] Kristoffersen ES, Harper CE, Vetvik KG, Zarnovicky S, Hansen JM, Faiz KW (2020). Incidence and mortality of cerebral venous thrombosis in a Norwegian population. Stroke.

[12] Ruuskanen JO, Kytö V, Posti JP, Rautava P, Sipilä JOT (2021). Cerebral venous thrombosis: Finnish nationwide trends. Stroke.

[13] Douxfils J, Favresse J, Dogné JM, Lecompte T, Susen S, Cordonnier C, Lebreton A, Gosselin R, Sié P, Pernod G, Gruel Y, Nguyen P, Vayne C, Mullier F (2021). Hypotheses behind the very rare cases of thrombosis with thrombocytopenia syndrome after SARS-CoV-2 vaccination. Thromb Res.

[14] Schultz NH, Sørvoll IH, Michelsen AE, Munthe LA, Lund-Johansen F, Ahlen MT, Wiedmann M, Aamodt AH, Skattør TH, Tjønnfjord GE, Holme PA (2021). Thrombosis and thrombocytopenia after ChAdOx1 nCoV-19 vaccination. N Engl J Med.

[15] Scully M, Singh D, Lown R, Poles A, Solomon T, Levi M, Goldblatt D, Kotoucek P, Thomas W, Lester W (2021). Pathologic antibodies to platelet factor 4 after ChAdOx1 nCoV-19 vaccination. N Engl J Med.

[16] Mehta PR, Apap Mangion S, Benger M, Stanton BR, Czuprynska J, Arya R, Sztriha LK (2021). Cerebral venous sinus thrombosis and thrombocytopenia after COVID-19 vaccination - A report of two UK cases. Brain Behav Immun.

[17] Greinacher A, Selleng K, Warkentin TE (2017). Autoimmune heparin-induced thrombocytopenia. J Thromb Haemost.

[18] Brodard J, Kremer Hovinga JA, Fontana P, Studt JD, Gruel Y, Greinacher A (2021). COVID-19 patients often show high-titer non-platelet-activating anti-PF4/heparin IgG antibodies. J Thromb Haemost.

[19] Furie KL, Cushman M, Elkind MSV, Lyden PD, Saposnik G (2021). Diagnosis and management of cerebral venous sinus thrombosis with vaccine-induced immune thrombotic thrombocytopenia. Stroke.

[20] Ferro JM, Aguiar de Sousa D (2019). Cerebral venous thrombosis: an update. Curr Neurol Neurosci Rep.

[21] Lum M, Tsiouris AJ (2020). MRI safety considerations during pregnancy. Clin Imaging.

[22] Bourguignon A, Arnold DM, Warkentin TE, Smith JW, Pannu T, Shrum JM, Al Maqrashi ZAA, Shroff A, Lessard MC, Blais N, Kelton JG, Nazy I (2021). Adjunct immune globulin for vaccine-induced immune thrombotic thrombocytopenia. N Engl J Med.

[23] Graf T, Thiele T, Klingebiel R, Greinacher A, Schäbitz WR, Greeve I (2021). Immediate high-dose intravenous immunoglobulins followed by direct thrombin-inhibitor treatment is crucial for survival in Sars-Covid-19-adenoviral vector vaccine-induced immune thrombotic thrombocytopenia VITT with cerebral sinus venous and portal vein thrombosis. J Neurol.

[24] Hinz P, Lubenow N, Ekkernkamp A, Greinacher A (2003). Informed consent on heparin-induced thrombocytopenia during thrombosis prophylaxis. A pilot study including 460 patients. Dtsch Med Wochenschr (1946).

[25] Patriquin CJ, Laroche V, Selby R, Pendergrast J, Barth D, Côté B, Gagnon N, Roberge G, Carrier M, Castellucci LA, Scarvelis D, Mack JP (2021). Therapeutic plasma exchange in vaccine-induced immune thrombotic thrombocytopenia. N Engl J Med.

[26] Coutinho JM, Zuurbier SM, Bousser MG, Ji X, Canhão P, Roos YB, Crassard I, Nunes AP, Uyttenboogaart M, Chen J, Emmer BJ, Roosendaal SD, Houdart E, Reekers JA, van den Berg R, de Haan RJ, Majoie CB, Ferro JM, Stam J (2020). Effect of endovascular treatment with medical management vs standard care on severe cerebral venous thrombosis: the TO-ACT randomized clinical trial. JAMA Neurol.

[27] Gessler F, Schmitz AK, Dubinski D, Bernstock JD, Lehmann F, Won SY, Wittstock M, Güresir E, Hadjiathanasiou A, Zimmermann J, Miesbach W, Freiman T, Vatter H, Schuss P (2021). Neurosurgical considerations regarding decompressive craniectomy for intracerebral hemorrhage after SARS-CoV-2-vaccination in vaccine induced thrombotic thrombocytopenia-VITT. J Clin Med.

[28] Walter U, Fuchs M, Grossmann A, Walter M, Thiele T, Storch A, Wittstock M (2021). Adenovirus-vectored COVID-19 vaccine-induced immune thrombosis of carotid artery: a case report. Neurology.

[29] Cuker A (2016). Management of the multiple phases of heparin-induced thrombocytopenia. Thromb Haemost.

[30] Ferro JM, Coutinho JM, Dentali F, Kobayashi A, Alasheev A, Canhão P, Karpov D, Nagel S, Posthuma L, Roriz JM, Caria J, Frässdorf M, Huisman H, Reilly P, Diener HC (2019). Safety and efficacy of dabigatran etexilate vs dose-adjusted warfarin in patients with cerebral venous thrombosis: a randomized clinical trial. JAMA Neurol.

[31] Walter U, Volmer E, Wittstock M, Storch A, Weber MA, Großmann A (2021). Cerebral venous sinus thrombosis after COVID-19 vaccination: neurological and radiological management. Radiologe.

