# Supplementary figures and images for: Cerebral venous sinus thrombosis after adenovirus-vectored COVID-19 vaccination: review of the neurological-neuroradiological procedure
Source: Neuroradiology. 2022 Feb 19;64(5):865–74. doi: 10.1007/s00234-022-02914-z (PMC8929723; doi:10.1007/s00234-022-02914-z)

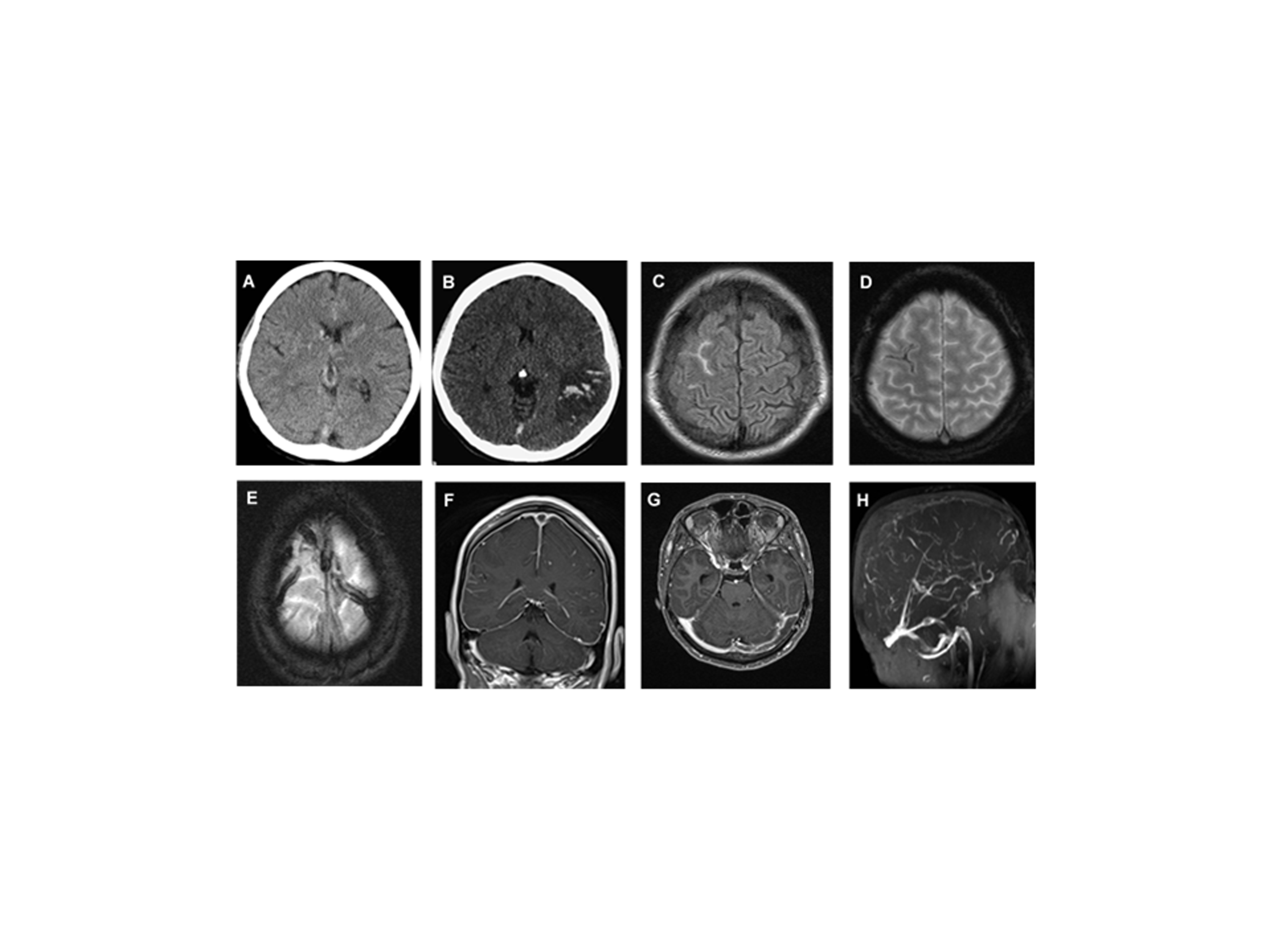

Supplement: Supplementary file 1 — Summary of pathognomonic signs of sinus and cerebral venous thrombosis on CT and MRI. A, hyperdense internal cerebral veins on CT scan; B, atypical bleeding on CT scan; C, sulcal SAB, hyperintense in FLAIR, which are hypointense in T2*-weighted images (D); E, blooming of the bridging veins in T2*; “cord sign” in CE-images (F, G); H, missing venous and sinus contrast in CE-MRI. (PNG 837 kb) [file 234_2022_2914_Fig5_ESM.png]

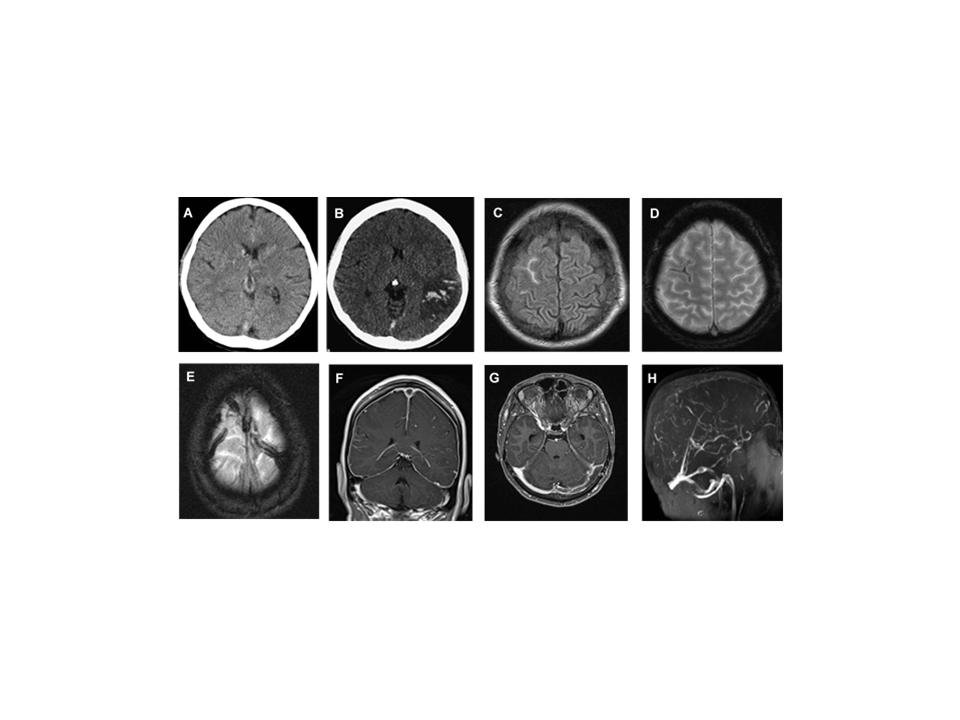

Supplement: Supplementary file 2 — High resolution image (TIF 260 kb) [file 234_2022_2914_MOESM1_ESM.tif]
