# Supplementary material for: Cerebral venous sinus thrombosis after adenovirus-vectored COVID-19 vaccination: review of the neurological-neuroradiological procedure
Source: Neuroradiology. 2022 Feb 19;64(5):865–74. doi: 10.1007/s00234-022-02914-z (PMC8929723; doi:10.1007/s00234-022-02914-z)
Supplement: Supplementary file 3 — Proposed protocol for MRI in case of suspicion of vaccine-induced CVST (Siemens Avanto; 1.5 T). DWI diffusion weighted imaging (diffusions weighted sequence, B1000), FLAIR fluid attenuated inversion recovery, FOV field of view, CM contrast media, MIP maximum intensity projection, MPR multiplanar reconstruction, CE-MRA contrast enhanced MR-angiography, PC phase contrast (phase-contrast-angiography), TR repetition time, TE echo time (DOCX 14 kb) [file 234_2022_2914_MOESM2_ESM.docx]

**Supplementary Table S1**

Proposed protocol for MRI in case of suspicion of vaccine-induced CVST (Siemens Avanto; 1.5 T)

|  | **unenhanced** | | | | | **With**  **contrast media** | |
| --- | --- | --- | --- | --- | --- | --- | --- |
| Weighting | T2w | T2w | T2w | T2w | T1w | T1w | T1w |
| Sequence designation | DWI | PC 3D | FLAIR | T2*w | T1w MPR | CE-MRA | T1w MPR post CM |
| TR (ms) | 4100 | 54,20 | 7000 | 908 | 1120 | 2.97 | 1120 |
| TE (ms) | 89 | 8.68 | 98 | 25.7 | 3 | 1.15 | 3 |
| Flip angle (°) | - | 150 | 150 | 20 | 8 | 30 | 8 |
| TI (ms) | - | - | 2500 | - | 900 | - | 900 |
| FOV (mm) | 230 x 230 | 120 x 120 | 230 x 230 | 230 x 230 | 230 x 230 | 270 x 270 | 230 x 230 |
| Percent Phase FOV (%) | 75 | 757 | 75 | 78,1 | 75 | 81.3 | 75 |
| Slice thickness (mm) | 5.0 | 2.0 | 5.0 | 5.0 | 3.0 | 3.0 | 3.0 |
| Layer orientation | tra | 3D MPR + MIP | sag,  tra and cor | tra | tra,  sag and cor | 3D MPR + MIP | tra,  sag and cor |
| Acquisition time (min) | 02:09 | 06:55 | 02:22 | 02:40 | 03:35 | 01:50 | 03:35 |

*DWI, diffusion weighted imaging (diffusions weighted sequence, B1000); FLAIR, fluid attenuated inversion recovery; FOV, field of view; CM, contrast media; MIP, maximum intensity projection; MPR, multiplanar reconstruction; CE-MRA, contrast enhanced MR-angiography; PC, phase contrast (phase-contrast-angiography); TR, repetition time; TE, echo time*
