# Supplementary material for: Cerebral venous sinus thrombosis after adenovirus-vectored COVID-19 vaccination: review of the neurological-neuroradiological procedure
Source: Neuroradiology. 2022 Feb 19;64(5):865–74. doi: 10.1007/s00234-022-02914-z (PMC8929723; doi:10.1007/s00234-022-02914-z)
Supplement: Supplementary file 4 — Proposed protocol for MRI in case of suspicion of vaccine-induced CVST (Siemens Magnetom Vida; 3.0 T). SWI susceptibility weighted imaging, FLAIR fluid attenuated inversion recovery, FOV field of view, CM contrast media, MIP maximum intensity projection, MPR multiplanar reconstruction, MRA MR-angiography, TWIST time-resolved angiography with interleaved stochastic trajectories, PC phase contrast (phase-contrast-angiography), TR repetition time, TE echo time (DOCX 14 kb) [file 234_2022_2914_MOESM3_ESM.docx]

**Supplementary Table S2**

Proposed protocol for MRI in case of suspicion of vaccine-induced CVST (Siemens Magnetom Vida; 3.0 T)

|  | **unenhanced** | | | | | **With**  **contrast media** | |
| --- | --- | --- | --- | --- | --- | --- | --- |
| Weighting | T1w | T2w | T2w | T2w | T2w | T1w | T1w |
| Sequence designation | T1w MPR | 3D FLAIR  (T2 space darkfluid) | resolve | T2*w / SWI | Flow PC3D | MRA (TWIST) | T1w MPR post CM |
| TR (ms) | 2100 | 7000 | 6350 | 800/ 30 | 63,5 | 2,26 | 2100 |
| TE (ms) | 2.46 | 393 | 59.99 | 20 | 6.68 | 0.92 | 2.46 |
| TI ( ms) | 900 | 2000 | - | - | - | - | 900 |
| Flip angle (°) | 8 | T2 var | 180 | 20/15 | 12 | 17 | 8 |
| FOV (mm) | 230 | 250 | 230 | 230 | 220 | 256 | 230 |
| Percent Phase FOV (%) | 75 | 100 | 100 | 75 | 100 | 100 | 75 |
| Slice thickness (mm) | 0.9 | 1 | 4 | 4/1.5 | 0.9 | 1 | 0.9 |
| Layer orientation | tra  (sag, cor  reconstruction) | 3D MPR + MIP | tra | tra | 3D MPR  +MIP | 3D MPR + MIP | tra  (sag, cor  reconstruction) |
| Acquisition time (min) | 3:46 | 5:52 | 3:31 | 2:26/2:57 | 7:07 | 1:32 | 3:46 |

*SWI, susceptibility weighted imaging; FLAIR, fluid attenuated inversion recovery; FOV, field of view; CM, contrast media; MIP, maximum intensity projection; MPR, multiplanar reconstruction; MRA, MR-angiography; TWIST*, *time-resolved angiography with interleaved stochastic trajectories; PC, phase contrast (phase-contrast-angiography); TR, repetition time; TE, echo time*
