# Supplementary material for: Cerebral venous sinus thrombosis after adenovirus-vectored COVID-19 vaccination: review of the neurological-neuroradiological procedure
Source: Neuroradiology. 2022 Feb 19;64(5):865–74. doi: 10.1007/s00234-022-02914-z (PMC8929723; doi:10.1007/s00234-022-02914-z)
Supplement: Supplementary file 5 — Signal of thrombosis in MRI depending on thrombus age (modified [17]). CM contrast media, PCA phase contrast angiography, SWI susceptibility-weighted imaging, TOF time-of-flight angiography (DOCX 12 kb) [file 234_2022_2914_MOESM4_ESM.docx]

**Supplementary Table S3**

Signal of thrombosis in MRI depending on thrombus age (modified [17]).

| **Sequence** | **acute thrombosis** | **1 week old thrombosis** | **2 to 6 weeks old thrombosis** |
| --- | --- | --- | --- |
| T1w unenhanced | Iso- hypointense | Hyperintense | hyperintense |
| T2w unenhanced | Hypointense | Hypointense | hyperintense |
| SWI/ T2*w | Blooming | Blooming | Blooming |
| Venous PCA | No flow signal | No flow signal | No flow signal |
| Venous TOF | Central sparing | „Pseudo flow“ | „Pseudo flow“ |
| T1w 3D post-CM | Central sparing | „Pseudo enhancement“ | „Pseudo enhancement“ |

*CM, contrast media; PCA, phase contrast angiography; SWI, susceptibility –weighted imaging, TOF, Time-of-flight- angiography*
